# A Candidate Gene Approach Identifies the *TRAF1*/*C5* Region as a Risk Factor for Rheumatoid Arthritis

**DOI:** 10.1371/journal.pmed.0040278

**Published:** 2007-09-18

**Authors:** Fina A. S Kurreeman, Leonid Padyukov, Rute B Marques, Steven J Schrodi, Maria Seddighzadeh, Gerrie Stoeken-Rijsbergen, Annette H. M van der Helm-van Mil, Cornelia F Allaart, Willem Verduyn, Jeanine Houwing-Duistermaat, Lars Alfredsson, Ann B Begovich, Lars Klareskog, Tom W. J Huizinga, Rene E. M Toes

**Affiliations:** 1 Department of Rheumatology, Leiden University Medical Centre, Leiden, The Netherlands; 2 Rheumatology Unit, Department of Medicine, Karolinska Institute at Karolinska Hospital, Stockholm, Sweden; 3 Celera, Alameda, California, United States of America; 4 Department of Immunohaematology and Bloodbank, Leiden University Medical Center, Leiden, The Netherlands; 5 Department of Medical Statistics, Leiden University Medical Centre, Leiden, The Netherlands; 6 Institute of Environmental Medicine, Karolinska Institute, Stockholm, Sweden; Kings College London, United Kingdom

## Abstract

**Background:**

Rheumatoid arthritis (RA) is a chronic autoimmune disorder affecting ∼1% of the population. The disease results from the interplay between an individual's genetic background and unknown environmental triggers. Although human leukocyte antigens (HLAs) account for ∼30% of the heritable risk, the identities of non-HLA genes explaining the remainder of the genetic component are largely unknown. Based on functional data in mice, we hypothesized that the immune-related genes *complement component 5 (C5)* and/or *TNF receptor-associated factor 1 (TRAF1),* located on Chromosome 9q33–34, would represent relevant candidate genes for RA. We therefore aimed to investigate whether this locus would play a role in RA.

**Methods and Findings:**

We performed a multitiered case-control study using 40 single-nucleotide polymorphisms (SNPs) from the *TRAF1* and *C5 (TRAF1/C5)* region in a set of 290 RA patients and 254 unaffected participants (controls) of Dutch origin. Stepwise replication of significant SNPs was performed in three independent sample sets from the Netherlands (*n*_cases/controls_ = 454/270), Sweden (*n*_cases/controls_ = 1,500/1,000) and US (*n*_cases/controls_ = 475/475). We observed a significant association (*p* < 0.05) of SNPs located in a haplotype block that encompasses a 65 kb region including the 3′ end of *C5* as well as *TRAF1*. A sliding window analysis revealed an association peak at an intergenic region located ∼10 kb from both *C5* and *TRAF1*. This peak, defined by SNP14/rs10818488, was confirmed in a total of 2,719 RA patients and 1,999 controls (odds ratio_common_ = 1.28, 95% confidence interval 1.17–1.39, *p*_combined_ = 1.40 × 10^−8^) with a population-attributable risk of 6.1%. The A (minor susceptibility) allele of this SNP also significantly correlates with increased disease progression as determined by radiographic damage over time in RA patients (*p* = 0.008).

**Conclusions:**

Using a candidate-gene approach we have identified a novel genetic risk factor for RA. Our findings indicate that a polymorphism in the *TRAF1/C5* region increases the susceptibility to and severity of RA, possibly by influencing the structure, function, and/or expression levels of *TRAF1* and/or *C5*.

## Abstract 2

Using a candidate-gene approach, Rene Toes and colleagues identified a novel genetic risk factor for rheumatoid arthritis in the*TRAF1/C5* region.

## Editors' Summary

**Background.:**

Rheumatoid arthritis is a very common chronic illness that affects around 1% of people in developed countries. It is caused by an abnormal immune reaction to various tissues within the body; as well as affecting joints and causing an inflammatory arthritis, it can also affect many other organs of the body. Severe rheumatoid arthritis can be life-threatening, but even mild forms of the disease cause substantial illness and disability. Current treatments aim to give symptomatic relief with the use of simple analgesics, or anti-inflammatory drugs. In addition, most patients are also treated with what are known as disease-modifying agents, which aim to prevent joint damage. Rheumatoid arthritis is known to have a genetic component. For example, an association has been shown with the part of the genome that contains the human leukocyte antigens (HLAs), which are involved in the immune response. Information on other genes involved would be helpful both for understanding the underlying cause of the disease and possibly for the discovery of new treatments.

**Why Was This Study Done?:**

Previous work in mice that have a disease similar to human rheumatoid arthritis has identified a number of possible candidate genes. One of these genes, *complement component 5 (C5)* is involved in the complement system—a primitive system within the body that is involved in the defense against foreign molecules. In humans the gene for C5 is located on Chromosome 9 close to another gene involved in the inflammatory response, *TNF receptor-associated factor 1 (TRAF1)*. A preliminary study in humans of this region had shown some evidence, albeit weak, to suggest that this region might be associated with rheumatoid arthritis. The authors set out to look in more detail, and in a larger group of individuals, to see if they could prove this association.

**What Did the Researchers Do and Find?:**

The researchers took 40 genetic markers, known as single-nucleotide polymorphisms (SNPs), from across the region that included the *C5* and *TRAF1* genes. SNPs have each been assigned a unique reference number that specifies a point in the human genome, and each is present in alternate forms so can be differentiated. They compared which of the alternate forms were present in 290 patients with rheumatoid arthritis and 254 unaffected participants of Dutch origin. They then repeated the study in three other groups of patients and controls of Dutch, Swedish, and US origin. They found a consistent association with rheumatoid arthritis of one region of 65 kilobases (a small distance in genetic terms) that included one end of the *C5* gene as well as the *TRAF1* gene. They could refine the area of interest to a piece marked by one particular SNP that lay between the genes. They went on to show that the genetic region in which these genes are located may be involved in the binding of a protein that modifies the transcription of genes, thus providing a possible explanation for the association. Furthermore, they showed that one of the alternate versions of the marker in this region was associated with more aggressive disease.

**What Do These Findings Mean?:**

The finding of a genetic association is the first step in identifying a genetic component of a disease. The strength of this study is that a novel genetic susceptibility factor for RA has been identified and that the overall result is consistent in four different populations as well as being associated with disease severity. Further work will need to be done to confirm the association in other populations and then to identify the precise genetic change involved. Hopefully this work will lead to new avenues of investigation for therapy.

**Additional Information.:**

Please access these Web sites via the online version of this summary at http://dx.doi.org/10.1371/journal.pmed.0040278.

• Medline Plus, the health information site for patients from the US National Library of Medicine, has a page of resources on rheumatoid arthritis

• The UK's National Health Service online information site has information on rheumatoid arthritis

• The Arthritis Research Campaign, a UK charity that funds research on all types of arthritis, has a booklet with information for patients on rheumatoid arthritis

• Reumafonds, a Dutch arthritis foundation, gives information on rheumatoid arthritis (in Dutch)

• Autocure is an initiative whose objective is to transform knowledge obtained from molecular research into a cure for an increasing number of patients suffering from inflammatory rheumatic diseases

• The European league against Rheumatism, an organisation which represents the patient, health professionals, and scientific societies of rheumatology of all European nations

## Introduction

Rheumatoid arthritis (RA) is characterized by chronic inflammation and destruction of the synovial joints leading to progressive joint damage and disability. The disease has a complex etiology, including a wide spectrum of clinical manifestations, variability in disease severity and/or progression, and differential response to a range of therapies. This heterogeneous phenotype suggests the involvement of both environmental and genetic factors [1], where the genetic component of RA has been estimated to be between 50%–60% [2,3]. Identification of disease-associated genes is important as it will guide our understanding of the biological pathways underlying polygenic diseases and the development of potential novel therapeutic targets.

The most prominent genetic association in RA is confined to the *human leukocyte antigen (HLA)* locus. Although this association has been known for almost 30 years, and although the underlying mechanism is still not understood, it has been replicated in multiple studies [2,4]. The identification of RA-associated genes outside of the *HLA* region, however, has been a challenge. Recently one such gene, *protein tyrosine phosphatase, non-receptor type 22 (lymphoid) (PTPN22)*, was identified in the first step of a large genetic-association study utilizing putative functional SNPs [5]. The gene product encoded by *PTPN22* is, like the *HLA* locus, involved in T cell-mediated immune responses. However, other immune components are also thought to play a pivotal role in RA, as demonstrated by the beneficial effects of treatment with agents that block proinflammatory cytokines, such as tumor necrosis factor α (TNFα) [6]. Moreover, in several experimental animal models for RA, innate immune responses mediated by a diversity of players have been implicated in arthritis. In this respect, a prominent role for the complement system has been identified as mice deficient in complement factors are resistant to arthritis, and as it has been shown that targeting *complement component 5 (C5)* by antibodies prevents the onset of arthritis and reduces the clinical severity in mouse models for arthritis [7,8]. Likewise, the observation that high levels of C5a*,* a potent chemoattractant, are found in synovial fluid of RA patients combined with the fact that *C5a receptor*-deficient mice are also resistant to arthritis induction, indicate a central role for these mediators in arthritis [9,10]. A genome scan of mice that were or were not susceptible to antibody-induced arthritis revealed that the main genetic influence detected in this model maps to the *C5* region [11].

These functional data in mice inspired us to hypothesize that the *C5* region would be a contributing factor in RA. Therefore, we searched for further evidence by first addressing the question of whether any genetic indications exist that implicate the involvement of this region in RA. A conventional linkage study using microsatellite markers identified a linkage peak in the vicinity of the *C5* region [12]. Although this study provided weak evidence for linkage (logarithm of the odds score, LOD 1.8), it further boosted our interest in this region. *C5* is located next to *TNF receptor-associated factor 1 (TRAF1)*, an essential effector of the TNF signaling cascade. Since TNF blockade represents a powerful intervention in both mice and humans for the treatment of arthritis, it provided an additional rationale to explore this genetic region encoding *C5* and *TRAF1,* which are adjacent to each other on Chromosome 9q33–34. We therefore sought to investigate whether these candidate genes, which are important immune mediators, would play a role in RA.

## Methods

### Study Populations

All RA patients in all sets in this study met the American College of Rheumatology 1987 revised criteria for RA [13].

Sample set 1 cases consisted of 290 RA patients consecutively included from the out-patient clinic of the Leiden University Hospital in 1994 [14] and 254 controls randomly selected by the section Immunogenetics and Transplantation Immunology of Leiden University Medical Center, Leiden, The Netherlands (ITI).

Since 89% of the first set of RA patients were rheumatoid factor (RF) positive, we genotyped an independent sample set 2, which consisted of 454 RF-positive patients from two inception cohorts of early arthritis patients (EAC and BEST) and a second set of 270 randomly selected Dutch blood donors from ITI. Briefly, the EAC consists of patients included from 1993 onwards and originating from a health care region of about 400,000 inhabitants in the western part of The Netherlands. General practitioners were encouraged to refer patients directly when arthritis was suspected. Patients were included when the symptom duration was less than 2 y. Patients from the BEST study were recruited between March 2000 and August 2002 at 20 centers in the western part of The Netherlands. Patients had a maximum disease duration of 2 y, were at least 18 y of age, and had active disease (defined as ≥ six swollen joints, ≥ six tender joints, and either an erythrocyte sedimentation rate of ≥ 28 mm/h or a global health assessment score of ≥ 20 on a 100-mm visual analog scale, where 0 = best and 100 = worst). Only patients with a diagnosis of RA were included in the present study. These cohorts are further described in detail in other reports [15,16].

Sample set 3 consisted of 1,500 RA patients (70% RF-positive) and 1,000 unaffected participants (controls) from the Swedish EIRA study as previously described [17]. Briefly, RA patients and controls aged 18–70 y during May 1996 to December 2003 from a geographically defined area in the south and central regions of Sweden. Control participants were randomly selected from a continuously updated national population register, with consideration given to age, sex, and living area. If the selected control was not traceable, reported having RA, or refused to participate, a new control was selected using the same procedure.

Sample set 4, obtained from the Genomics Collaborative, (GCI), comprised 475 RF-positive RA patients and 475 individually matched controls from the US and has been described in detail elsewhere [5]. In brief, all case samples were from white North Americans who were RF-positive and whose condition met the 1987 American College of Rheumatology diagnostic criteria for RA. Control samples were taken from a pool of healthy white individuals with no medical history of RA. A single control was matched to each case on the basis of sex, age (±5 y), and ethnicity (grandparental country/region of origin).

Patients from sample sets 1 and 4 had considerably longer disease duration at inclusion (13.8 ± 10.1 y and 11.7 ± 10.0 y, respectively) as compared to patients from sample sets 2 and 3 (<3 y) (Table S1). All controls were healthy unrelated white individuals originating from the same geographical area as the patients. There was no overlap between cases and controls across all studies. All protocols and recruitments were approved by national and/or local institutional review boards, and informed written consent was obtained from all participants.

### SNP Selection and Genotyping

We chose 40 polymorphisms spanning *TRAF1*/*C5* and their flanking genes *PHD finger protein 19* (*PHF19*) and *centrosomal protein 110 kDa* (*CEP110)* for this study (Table 1). We selected haplotype tagging SNPs (htSNPs) from the International HapMap Project database (http://www.hapmap.org/index.html) as well as random SNPs from the University of California Santa Cruz database (http://genome.ucsc.edu/) to ascertain maximum haplotype information for each of the genes and intergenic regions that are likely to harbor regulatory regions. Chromosomal locations of the SNPs were extracted from SNPPER (http://snpper.chip.org/) Goldenpath hg17, dbSNP build 123. Genotyping across all studies was performed as described in detail in Protocol S1. Three of the 40 SNPs were excluded from further analysis after reviewing results from sample set 1, two of which (SNP13/rs4837803 and SNP27/rs10119768) were not polymorphic and one (SNP35/rs7856420) of which deviated from Hardy-Weinberg equilibrium.

**Table 1:**
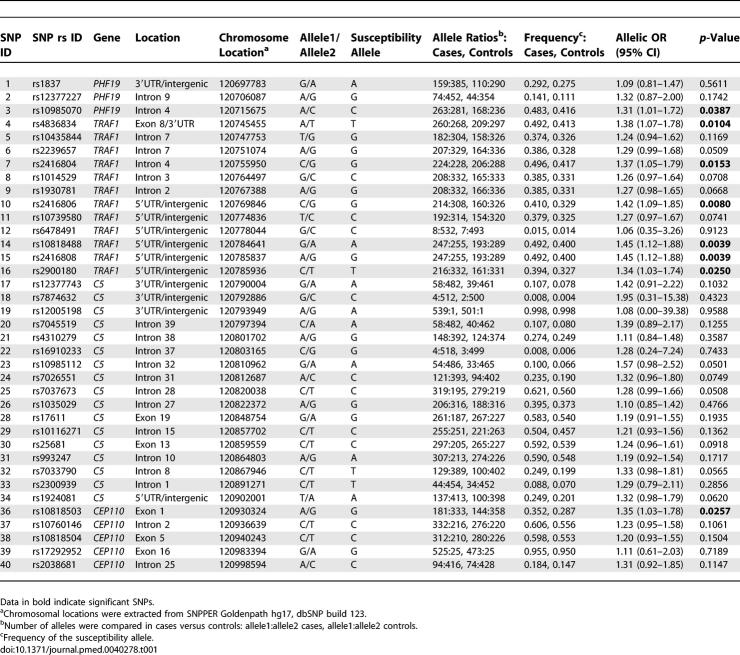
Association of Candidate Gene SNPs with RA

### Statistical Analysis

#### Single SNP and haplotype analysis.

Single SNP analysis and genetic model assessment was initially performed using SPSS version 12.0 (SPSS, http://www.spss.com/) in sample set 1. We did not find evidence of a recessive model for any SNPs. Since all SNPs were in Hardy-Weinberg equilibrium and adhered to the additive model of association, we performed further tests using allelic comparisons. Single- and multilocus allelic analyses were performed using Haploview version 3.32 (MIT, http://www.broad.mit.edu/mpg/haploview/) [18] with 40 SNPs in sample set 1 followed by six significant SNPs in sample set 2, the three most significant SNPs in sample set 3, and the single best-associating SNP in sample set 4. Odds ratios were calculated using Epi Info v6 (CDC, http://www.cdc.gov/epiinfo/). All *p*-values reported were two-sided. A *p*-value < 0.05 was considered significant. Based on the linkage disequilibrium (LD) structure in sample set 1, haplotype blocks were inferred under the algorithm of Gabriel et al. [19] in Haploview 3.32. To further minimize haplotypic uncertainty, we used the software TagSNPs version 1.0 (http://www-rcf.usc.edu/~stram/tagSNPs.html) to identify eight htSNPs from block 2 and ten htSNPs from block 3 (global *R* ^2^_h_ > 98%) [20]. The *R* ^2^_h_ coefficient is the squared correlation between the true haplotype count (number of copies of a haplotype) and the haplotype count predicted by TagSNPs. We chose htSNPs so that haplotypes were predicted with a global *R* ^2^_h_ value of 0.95 or above, indicating a high accuracy. Haplotype analyses using these htSNPs were performed in Haploview 3.32.

Global *p*-values for haplotype associations were calculated using the software Haplo.Stats version 1.2 (http://mayoresearch.mayo.edu/mayo/research/schaid_lab/software.cfm) used for estimating haplotype effects under the generalized linear model [21]. htSNPs from blocks 2 and 3 in sample set 1 were further investigated by sliding-window analysis in Haploview 3.32 to determine the basis of the associated haplotypes.

#### Combining datasets.

Odds ratios (OR)s from all sample sets were combined by the random model of the Cochran-Mantel-Haenszel test as implemented in EasyMA [22]. A Breslow-Day test of between-stage heterogeneity was also performed in EasyMA to test for consistency across sample sets [23]. We observed evidence of heterogeneity for SNP rs4836834 at *p* < 0.05 (Table S2).

#### Logistics regression.

Forward conditional logistics regression was performed using all six significant SNPs from sets 1 and 2 in SPSS 12.0. Genotypes were coded as categorical variables 0, 1, and 2 with the nonassociated genotype as reference. No evidence of a recessive effect was observed, as detailed in Table S3.

#### Population attributable risk.

Population-attributable risk (PAR) was calculated using the population prevalence of the exposure *(P)* and the relative risk associated with the exposure *(R),* as follows [24].

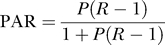



### Autoantibodies

Baseline laboratory parameters included IgM-RF (ELISA) and IgG-ACPA (ELISA, Immunoscan RA Mark2, Euro-Diagnostica, http://www.eurodiagnostica.com/). The cut-off for antibody positivity was set according to the manufacturer's instructions. Since baseline ACPA status was not available for most samples from the BEST study, we restricted our analysis to the EAC and EIRA cohorts. Autoantibody (RF and ACPA) and genotype status were available from 419 patients from the EAC (we additionally genotyped RF-negative and ACPA-negative patients in this cohort) and 1,395 patients from the EIRA study.

### Severity

Radiographs of hands and feet were scored at baseline and 2 y (*n* = 278) using the Sharp–van der Heijde method (erosions and joint space narrowing of hands and feet) [25]. Data was available from 193 A carriers (AA+AG) and 85 non-A carriers (GG) from the EAC cohort. The prevalence of either RF (A carriers 60.9%, non-A carriers 56.5%, *p* = 0.485) or anti-citrullinated protein antibodies (ACPA) (A carriers 60.7%, non-A carriers 60.8%, *p* = 0.986) is not significantly different between the two groups analyzed. Differences in means between groups were calculated using sharp scores adjusted for baseline with a two-sided nonparametric Mann-Whitney test.

### Transcription Factor Binding Sites

Transcription factor binding sites (TFBSs) were predicted using MAPPER (http://bio.chip.org/mapper/mapper-main), a platform that combines information from two well-known TFBS databases, TRANSFAC and JASPAR. The prediction is generated from a hidden Markov model and is based on experimentally determined binding sites in multiple genomes [26].

## Results

### The *TRAF1*/*C5* Region Associates with RA

To investigate whether the *TRAF1*/*C5* region on Chromosome 9q33–34 associates with RA, we selected a total of 40 polymorphisms spanning these candidates and their flanking genes for genotyping. Tagging SNPs as well as random SNPs were included to ascertain maximum haplotype information for each of the genes and to ensure coverage of intergenic regions which may harbor regulatory polymorphisms.

Single SNP analysis performed in the first set of RA patients (*n* = 290) and controls (*n* = 254) revealed significant association between six SNPs in the *TRAF1*/*C5* region (SNPs 4, 7, 10, 14, 15, and 16) and RA (*p* = 0.0104, 0.0153, 0.0080, 0.0039, 0.0039, and 0.0250, respectively) (Table 1). One SNP in *PHF19* (SNP3/rs10985070) and one in *CEP110* (SNP36/rs10818503) also showed moderate association with RA (*p* = 0.0387 and 0.0257, respectively). None of the SNPs investigated showed evidence of a recessive mode of association.

**Figure 1:**
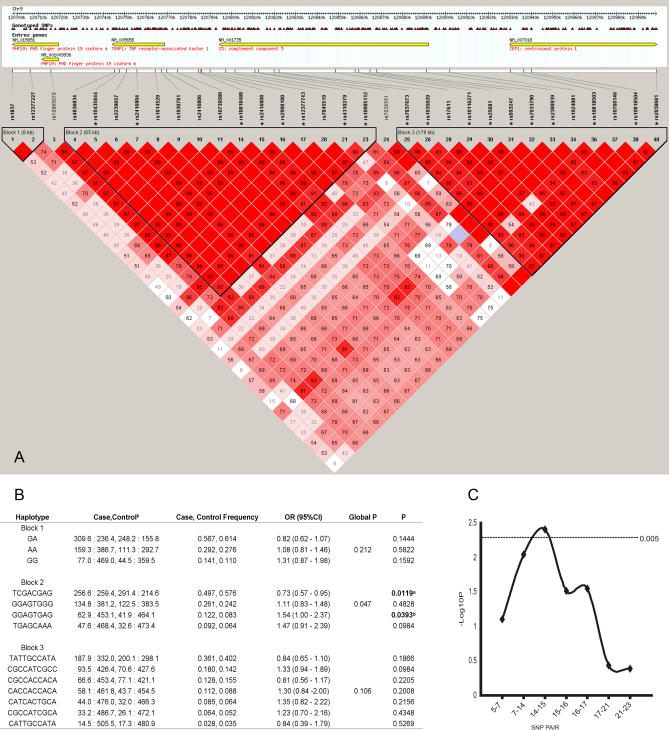
LD Structure and Haplotype Association across the *TRAF1/C5* Region in Sample Set 1 (290 RA Patients and 254 Controls) (A) Haplotype block structure was predicted on the basis of the strength of pairwise LD, which is presented as a 2×2 matrix; red represents very high LD *(D'),* white indicates absence of correlation between SNPs, and blue indicates high correlation with a low level of significance. SNPs that were chosen for haplotype analysis are indicated along the top by an asterisk. (B) Using htSNPs from each block, indicated by the asterisk in (A), haplotypes were inferred with a certainty of above 98% as represented by the *R* ^2^_h_ value. ^¥^Comparisons were made between one haplotype versus all others in cases and controls. ^a^The protective haplotype that is significantly less frequent in cases. ^b^The susceptible haplotype that is significantly more frequent in patients. (C) Sliding window of the susceptible haplotype using consecutive two-SNP combinations of the htSNPs reveals that SNP14 and SNP15 account for most of the association observed. For each of the SNP pairs we show the –log_10_ *p*-value. The dotted line indicates a nominal *p*-value of 0.005.

To delineate the haplotypic architecture, we estimated the underlying haplotype block structure of the 32 SNPs with minor allele frequency > 5% in the controls. We identified a potential recombination spot at SNP3/rs10985070 and SNP24/rs7026551 that divides the region into three inheritance blocks, blocks 1, 2, and 3. Block 1 is 8 kb and encompasses the 3′ end to intron 9 of *PHF19;* block 2 extends from *TRAF1* through 24 kb of intergenic sequence to exon 32 of *C5;* while block 3 (178 kb) contains the remainder of the *C5* gene and *CEP110* (Figure 1A). The LD structure in the Dutch population was similar to the structure reported by the International HapMap Project (unpublished data).

**Figure 2:**
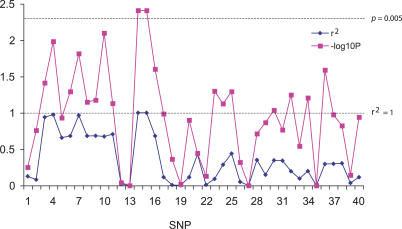
RA-Associated SNPs are Highly Linked to SNP14 Pairwise LD (*r* ^2^) between associated SNP14/rs10818488 and all other SNPs genotyped was calculated. For each of the SNPs listed along the x-axis we show the –log_10_ (y-axis) of the *p*-values for RA patients versus controls. Dotted lines indicate a nominal *p*-value of 0.005 and a maximal *r* ^2^ value of 1. In a logistics regression model, only SNP14/rs10818488 remained statistically significant (*p* = 6.16 × 10^−4^)

To further minimize haplotypic uncertainty and to identify the minimal combination of SNPs that provide maximum information content within each block, we scanned these two blocks independently using the software TagSNPs. In block 2 we identified a minimal set of eight htSNPs with a global *R* ^2^_h_ of 0.996, and in block 3 we found ten htSNPs with an *R* ^2^_h_ of 0.985, indicating that haplotypes can be inferred with >95% certainty. Haplotypes were predicted, and analyses from all blocks revealed that the association with RA was restricted to SNPs in block 2 (Figure 1B), as demonstrated by the global *p*-value of association (*p* < 0.05) and suggesting the possible involvement of *TRAF1* and/or the 3′ end of *C5*. Of the four common haplotypes capturing > 95% of the variation, two significantly associated haplotypes were observed, one increased in RA patients (susceptible haplotype^b^ *p* = 0.039), and one over-represented in controls (protective haplotype^a^ *p* = 0.012). By applying a two-marker sliding window analysis, we observed a significant peak centered on SNP14/rs10818488 and SNP15/rs2416808 (*p* = 0.0039) (Figure 1C). Using three- and five-marker windows did not alter the outcome, suggesting that the significance seen with the other *TRAF1* SNPs (Table 1) may be due to LD. To explore this possibility, we analyzed the *r* ^2^-values with respect to SNP14. We confirmed that the most significant SNPs, which are located in *TRAF1* and the adjacent intergenic region, are highly correlated with SNP14 (*r* ^2^ > 0.90) (Figure 2).

### Replication in Three Independent Sample Sets from The Netherlands, Sweden, and the US

Six tagging SNPs which were significant (*p* < 0.05) in the initial study were selected for replication in a fully independent set of Dutch cases and controls (sample set 2). Of these, only SNP14/rs10818488 and SNP15/rs2416808 were statistically significant (*p* < 0.05). Haplotype and a two-marker sliding window analysis localized the strongest region of association to SNP14 and SNP15, confirming the results from sample set 1 (unpublished data). Combined analyses of the data from sample sets 1 and 2 showed an even stronger association for SNP14 (OR 1.34, 95% confidence interval [CI] 1.13–1.58; *p* = 5.56 × 10^−4^) and SNP15 (OR 1.33, 95% CI 1.13–1.57; *p* = 6.65 × 10^−4^) (Table 2). Although the other four SNPs did not reach statistical significance in sample set 2, they were highly significant in the combined analysis (Table 2). To evaluate putative modes of inheritance, we calculated genotype-specific ORs in the combined dataset as detailed in Table S3. All SNPs were consistent with an additive model. On the basis of forward conditional logistics regression, SNP14 remained in the model with a heterozygote (AG) OR of 1.38 (95% CI 1.04–1.83, *p* = 0.027) and a homozygote (AA) OR of 2.06 (95% CI 1.42–2.98, *p* = 1.29 × 10^−3^).

**Table 2:**
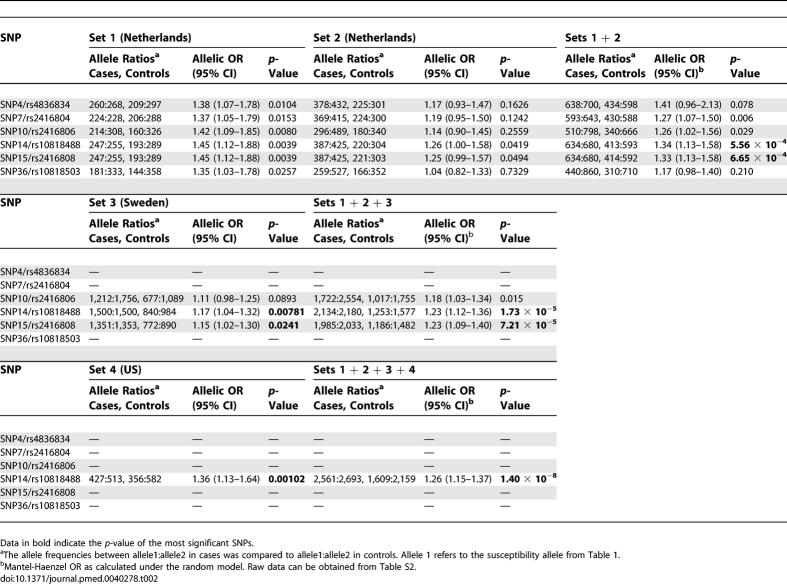
Association of Significant SNPs in Four Independent Sample Sets

Similar replication of three SNPs in a cohort of Swedish patients and controls (sample set 3) confirmed association with SNP14/rs10818488 (*p* = 0.0078) (Table 2). A combined analysis of patients and controls of European origin (Dutch and Swedish) with 2,244 RA patients and 1,524 controls (sample sets 1, 2, and 3) showed that the most significant associations could again be attributed to SNP14 (OR 1.24, 95% CI 1.11–1.38, *p* = 1.73 × 10^−5^) and SNP15/rs2416808 (OR 1.23, 95% CI 1.09–1.40, *p* = 7.21 × 10^−5^) (Table 2). Additionally, these findings were further replicated in a case-control sample set from the US. Since LD analysis in the original three sample sets showed SNP14 and SNP15 to be highly correlated (*r* ^2^ > 0.98) we genotyped only SNP14 and confirmed that the minor susceptibility allele was associated with RA risk (OR 1.36, 95% CI 1.13–1.64; *p* = 0.001) (Table 2). Combined analysis of SNP14 in all four independent sets (*n*_cases/controls_ = 2,719/1,999) yielded a highly significant association OR_common_=1.26, 95% CI 1.15–1.37, *p*_combined_ = 1.40 × 10^−8^) and a PAR of 6.1% (95% CI 4.0–8.5).

### Association with Autoantibody-Positive Disease

**Figure 3:**
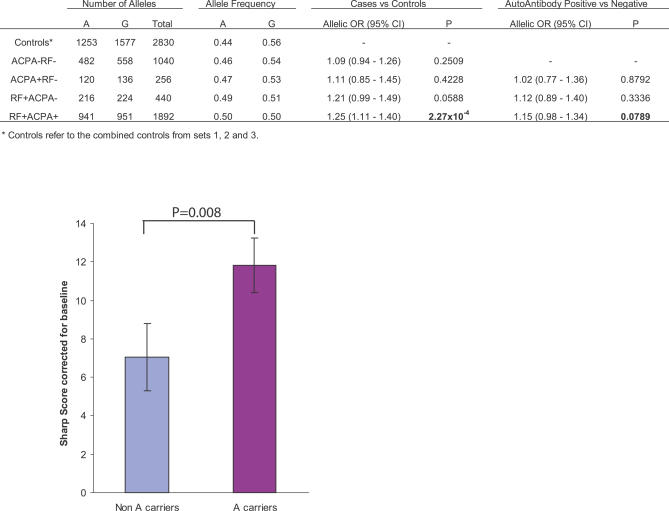
The A Allele of SNP14 Is Associated with Distinct Phenotypes of RA (Top, table) The frequency of the A allele of patients from whom baseline autoantibody status (ACPA and RF) were available was calculated. ORs and *p*-values were calculated between each subgroup and controls, and indicated a predominantly higher frequency of the A allele in ACPA- and RF-positive patients. (Bottom, bar graph) Progression of joint damage in Sharp–van der Heijde units (“Sharp score”) is higher in the presence of the minor A allele of SNP14. Radiological data of 193 A carriers and 85 non-A carriers were available, and differences between the groups were calculated based on disease severity after 2 y corrected for baseline.

RA is a heterogeneous disease with a considerable variation in phenotype as evidenced by the fact that some patients are autoantibody-positive whereas others are not. Antibodies to citrullinated protein antigens, called ACPAs, have gained much interest as current data suggest that ACPA-positive and negative RA may have different genetic risk factors [27]. To investigate whether the *TRAF1*/*C5* region is associated with a specific phenotype of RA, we next stratified patients for autoantibody status from whom baseline ACPA and RF measurements were available (*n* = 1,814) Interestingly, SNP14/rs10818488 mainly predisposes to autoantibody-positive disease when compared to controls (OR 1.25, 95% CI 1.11–1.40, *p* = 2.27 × 10^−4^) (Figure 3A). Although we also observed an increase in the frequency of the A allele in autoantibody-positive as compared to autoantibody-negative disease, this difference did not reach formal statistical significance (OR 1.15, 95% CI 0.98–1.34, *p* = 0.0789). These data therefore suggest that the current genetic risk factor may be predominant in the autoantibody-positive subset of RA patients.

### Association with Severity

Because the clinical course of RA can vary considerably ranging from nonerosive disease to rapidly progressive joint damage, we also analyzed whether the SNPs in the *TRAF1/C5* region were involved with RA progression. Annual X-rays of the hands and feet of patients were assigned Sharp–van der Heijde units, a combined score for bone erosions and joint space narrowing. Carriers of the minor susceptibility allele of SNP14/rs10818488 had an almost 2-fold higher severe disease course at 2 y after inclusion as compared to the non-A carriers (Figure 3B; mean ± SE score of A carriers/non-A carriers, 11.4 ± 1.4/7.1 ± 1.8; *p* = 0.008) indicating that the A allele predisposes not only to RA susceptibility, but also to severity.

### SNP14/rs10818488 Is Located in a TFBS

To investigate the potential functional effect of this SNP, we scanned for transcription factor binding sites using MAPPER [26]. The SNP14 susceptibility A allele encodes a potential binding site for EP300, a histone acetyl transferase that regulates transcription via chromatin remodeling. In the absence of this allele, the binding of EP300 to this region is predicted to be disrupted, potentially disturbing the epigenetic tag for transcriptional activation. We hypothesize that this putative transcription factor binding site may be involved in the regulation of the neighboring *TRAF1* and/or *C5* gene.

## Discussion

Using a candidate-gene approach, we identified the *TRAF1*/*C5* region on Chromosome 9q33–34 as a susceptibility and severity factor for RA. This region was also associated with RA in a large-scale genetic association study (Schrodi et al., unpublished data). It is, therefore, intriguing to see that these independent studies, in which the process leading to results differed, give similar results, and in doing so provide strong evidence for the *TRAF1/C5* region as a true RA-associated genetic variant. The recent genome-wide study performed by the Wellcome Trust failed to identify this region as a candidate for RA [28]. Although it is difficult to speculate why this region was not detected in the Wellcome Trust Case Control Consortium study, we do note that none of the SNPs showing strong association in our hands was genotyped by the Wellcome Trust. Additionally, in line with our finding that this genetic risk factor is predominant in the autoantibody-positive subgroup, substratifications of the specific RA phenotypes may be needed to detect significant association.

The protein encoded by *TRAF1* is a member of the TNF receptor-associated factor (TRAF) protein family, which associates with and mediates the signal transduction from various receptors of the TNF receptor superfamily, including the receptor for TNFα [29]. In addition to a direct role in TNFα signaling, *TRAF1* has also been implicated in the activation and proliferation of T cells [30] and is expressed ubiquitously by other cells of the immune system including monocytes and B cells [31]. It is therefore possible that TRAF1 could play a role in RA by aiding the maintenance of the proinflammatory environment. Likewise, studies have also shown that perpetuation of inflammation coincides with increased levels of the anaphylatoxin C5a in the synovial fluid of RA patients [9]. Further studies in mice identifying *C5* as a candidate gene and showing that *C5* deficiency results in lower incidence and less-severe disease course support the role of this gene in inflammation [7,11]. It is therefore likely that although the primary function of the complement system is to protect the host from microorganisms, a deregulated activity of its central component, *C5,* can play a substantial role in inflammatory diseases as well.

In order to capture the variation within these candidate genes and potential regulatory regions, we genotyped both SNPs that were intragenic and those located 5′ and 3′ of the genes. Interestingly, the strongest replicated association was observed with SNP14/rs10818488, which maps to an intergenic region ∼10 kb from both *TRAF1* and *C5* and is present in a TFBS, which may regulate the transcriptional activity of its neighboring genes. However, formal testing of all known variation within this locus, both genetic and biological, will be necessary to pinpoint the precise biological process that is altered by the RA-associated variant(s) present in this region.

We found a strong association of this region in all four independent sample sets which represent varying disease durations (<3 to >10 y) as well as a correlation with disease progression. More importantly, these phenotypic data on joint destruction not only indicate that the *TRAF1*/*C5* region predisposes to RA, but also suggest that within the RA population, patients harboring the minor susceptibility allele of SNP14/rs10818488 tend to experience a more severe disease course. Although the above findings most likely exclude the possibility of a spurious association, especially since each case group was assigned an ethnically and geographically matched control group, background levels of population stratification as described by Cardon et al. [32] may exist in the different populations under study. It is therefore conceivable that the slight variation in the observed effect between the four populations may partially be due to varying sample sizes and background levels of population admixture.

RA is a common complex disease that results from the interaction of multiple genetic variants, each with relatively low penetrance, with an array of environmental triggers [33]. In advance of a genetic profile that can accurately pinpoint individuals at risk, identification of these genetic variants can provide insight into the underlying mechanisms of disease and the specific pathways associated with disease induction and/or progression. Understanding the function of these common disease-associated variants will be important to identify potential targets for intervention strategies that could prove useful to all patients, whether or not they carry the disease-associated variant.

In summary, this study provides robust evidence from four independent sample sets (two of Dutch origin, one of Swedish descent, and one from the US) that genetic variants within the *TRAF1/C5* region are associated with RA, indicating a possible role for these immune-related genes in the biological process underlying RA disease pathogenesis.

### Limitations of This Study

Our study defines the *TRAF1/C5* as a novel genetic region present in the human genome that predisposes to RA. However, the causative variation (SNP) or the biological mechanism explaining this association is not yet known. Although it could be that the current identified polymorphism is causative, other proxies in high LD with this SNP could also be responsible for the observed association, an issue that can be addressed in more detailed by functional studies. Furthermore, although our data indicate a predominant association with autoantibody-positive disease, our study is underpowered to exclude the possibility that the *TRAF1/C5* region also predisposes to autoantibody-negative disease. By combining information obtained from other cohorts in which both the autoantibody and the *TRAF1/C5* status are known, this question should be resolved in the future. As it has been indicated that distinct genetic risk factors underlie either autoantibody-positive or autoantibody-negative disease, such additional information would provide more detailed knowledge on the genetic heterogeneity of these two distinct phenotypes of RA.

## Supporting Information

Protocol S1 Genotyping Methods (26 KB DOC)

Table S1 Clinical Characteristics of RA Patients from Four Independent Sample Sets (21 KB XLS)

Table S2 Mantel-Haenzel OR and P for Combined Sets (22 KB XLS)

Table S3 Genotypic Odds Ratios for the Most Significant SNPs in Sample Sets 1 and 2 (21 KB XLS)

Table S4 Primers and Conditions for PCR_RFLP (21 KB XLS)

### Accession Numbers

The GenBank (http://www.ncbi.nlm.nih.gov/sites/entrez?db=pubmed) accession numbers for the genes discussed in this article are *C5* (NM_001735); *CEP110* (NM_007018); *PHF19* (XM_045308); *TRAF1* (NM_005658). The ExPASy UniProtKB/Swiss-Prot (http://www.expasy.org/sprot/) accession number for EP300 is Q09472.

## Abbreviations

ACPA: anti-citrullinated protein antibody
CI: confidence interval
HLA: human leucocyte antigen
htSNP: haplotype tagging SNP
LD: linkage disequilibrium
OR: odds ratio
RA: rheumatoid arthritis
RF: rheumatoid factor
SNP: single-nucleotide polymorphism
TNF: tumor necrosis factor
