# Correction: A Candidate Gene Approach Identifies the *TRAF1/C5* Region as a Risk Factor for Rheumatoid Arthritis

**DOI:** 10.1371/journal.pmed.0040358

**Published:** 2007-12-27

**Authors:** Fina A. S Kurreeman, Leonid Padyukov, Rute B Marques, Steven J Schrodi, Maria Seddighzadeh, Gerrie Stoeken-Rijsbergen, Annette H. M van der Helm-van Mil, Cornelia F Allaart, Willem Verduyn, Jeanine Houwing-Duistermaat, Lars Alfredsson, Ann B Begovich, Lars Klareskog, Tom W. J Huizinga, Rene E. M Toes

Correction for:

Kurreeman FAS, Padyukov L, Marques RB, Schrodi SJ, Seddighzadeh M, et al. (2007) A Candidate Gene Approach Identifies the *TRAF1/C5* Region as a Risk Factor for Rheumatoid Arthritis. PLoS Med 4(9): e278. doi:10.1371/journal.pmed.0040278

In Table 1, the allele ratio in column eight (Allele Ratios^b^: Cases, Controls) refers to allele A: allele B and not allele1:allele2 as described in footnote b, with Allele A being the Susceptibility Allele as denoted in column seven.

The footnote should read:

^b^Number of alleles were compared in cases versus controls: allele A: allele B cases, allele A: allele B controls. Allele A refers to the susceptibility alleles as given in column seven.

