# Supplementary material for: A Candidate Gene Approach Identifies the TRAF1/C5 Region as a Risk Factor for Rheumatoid Arthritis
Source: PLoS Med. 2007 Sep 18;4(9):e278. doi: 10.1371/journal.pmed.0040278 (PMC1976626; doi:10.1371/journal.pmed.0040278)
Supplement: Supplementary file 1 [file pmed.0040278.sd001.doc]

**Genotyping methods**

Cases and controls from *sample set 1* and cases from *sample set 2* were genotyped using the MassArray matrix-assisted laser desorption ionisation-time-of-flight mass spectrometry, according to the protocols recommended by the manufacturer (Sequenom, San Diego, California, USA). SpectroCaller software supplied by the manufacturer was used to automatically call the genotypes. Each 384-wells plate consisted of 8 positive controls (CEPH DNA) and 8 negative controls all of which had to be positive and negative respectively. Clusters were evaluated, all doubtful calls were checked, and after manually evaluating their spectra, they were accepted, recalled or rejected. 10% of the genotypes were performed in duplicate with an error rate less than 1%. If the CEPH DNA was genotyped for the SNPs in hapmap, genotypes were compared.

Controls in *sample set 2* were genotyped for six SNPs using the PCR-RFLP method. The PCR for rs4837813 genotyping was carried out using 10ng of genomic DNA in a reaction tube containing 1 unit Go*Taq* polymerase with the green GoTaq-buffer supplied by the manufacturer (Promega Corporation, Madison, WI, U.S.A) and 2.5mM MgCl2, 0.25mM of each dNTP (Invitrogen, Life Technologies, Gaithersburg, MD, U.S.A.) and 0.25µM of each primer in a 30-µl reaction. Primers were sense 5’-GCC CCA CAT ATA CCC ACC ATT TAA TTC AAG AAA AAG GAA C-3’ and antisense 5’-ACC AAC TGC ATT TGA TGC C-3’. The DNA was denatured at 94°C for 5 min and cycling conditions were set at 94°C for 15 sec, 62°C for 15 sec and 72°C for 30 sec for 37 cycles and a final cycle of extension at 72°C for 7 min in a thermocycler (Applied Biosystems 9700). The digestion was carried out with 10µl PCR-product, 2 units per tube of *Nla*IV, 10µg/ml bovine serum albumin and buffer as supplied by the manufacturer (MBI Fermentas, St.Leon-Rot, Germany) for 3 hours at 37°C. The digested fragments were separated by 4% agarose-gel electrophoresis and visualized by ethidium bromide staining and ultraviolet transillumination. The genotype was determined by the size of the fragments generated (194bp for the T allele, and 156 + 38bp for the C allele). The conditions for all other SNPs were essentially the same. The protocol for each SNP is outlined in Supplementary Table 4. Each 96-wells plate consisted of 4 positive and 4 negative controls. 10% of the samples were run in duplicate with no errors detected. The frequency of the second control set was similar to the first control set indicating that there was no method specific skewing of genotypes.

Genotyping of the three SNPs (rs2416806, rs10818488, rs2416808) in the EIRA study was performed using pre-designed Taqman-allelic-discrimination-assay (Applied Biosystems) according to the manufacturer’s protocol. Each plate consisted of 9 negative controls to check for contaminations. No discrepancy was detected. The data were manually curated and the clusters evaluated. Doubtful genotype calls were rejected. There were no specific fall-outs in any specific groups.

Genotyping of rs10818488 in the US *sample set* was done using allele specific kinetic PCR as previously described [1]. The data were hand-curated without knowledge of case-control status before statistical analysis with a 99% genotyping success rate and previous analyses suggest a genotyping accuracy of >99%.

References

1.	Begovich AB, Carlton VE, Honigberg LA, Schrodi SJ, Chokkalingam AP et al. (2004) A missense single-nucleotide polymorphism in a gene encoding a protein tyrosine phosphatase (PTPN22) is associated with rheumatoid arthritis. Am J Hum Genet 75: 330-337.
